# Kinetic Investigation on Tetrakis(4-Sulfonatophenyl)Porphyrin J-Aggregates Formation Catalyzed by Cationic Metallo-Porphyrins

**DOI:** 10.3390/molecules25235742

**Published:** 2020-12-05

**Authors:** Ilaria Giuseppina Occhiuto, Roberto Zagami, Mariachiara Trapani, Maria Angela Castriciano, Andrea Romeo, Luigi Monsù Scolaro

**Affiliations:** 1 Dipartimento di Scienze Chimiche, Biologiche, Farmaceutiche ed Ambientali, University of Messina and C.I.R.C.M.S.B. V.le F. Stagno D’Alcontres, 31-98166 Messina, Italy; ilaria.occhiuto@gmail.com (I.G.O.); anromeo@unime.it (A.R.); 2 CNR-ISMN Istituto per lo Studio dei Materiali Nanostrutturati c/o Dipartimento di Scienze Chimiche, Biologiche, Farmaceutiche ed Ambientali, University of Messina, V.le F. Stagno D’Alcontres, 31-98166 Messina, Italy; roberto.zagami@ismn.cnr.it (R.Z.); mariachiara.trapani@cnr.it (M.T.); maria.castriciano@cnr.it (M.A.C.)

**Keywords:** J-aggregates, aggregation kinetics, chiral supramolecular assemblies, symmetry breaking

## Abstract

Under mild acidic conditions, various metal derivatives of tetrakis(4-*N*-methylpyridinium)porphyrin (gold(III), AuT_4_; cobalt(III), CoT_4_; manganese(III), MnT_4_ and zinc(II), ZnT_4_) catalytically promote the supramolecular assembling process of the diacid 5,10,15,20-tetrakis(4-sulfonatophenyl)porphyrin (H_2_TPPS_4_) into J-aggregates. The aggregation kinetics have been treated according to a well-established model that involves the initial formation of a critical nucleus containing *m* porphyrin units, followed by autocatalytic growth, in which the rate evolves as a power of time. An analysis of the extinction time traces allows to obtain the rate constants for the auto-catalyzed pathway, *k_c_*, and the number of porphyrins involved in the initial seeding. The aggregation kinetics have been investigated at fixed H_2_TPPS_4_ concentration as a function of the added metal derivatives MT_4_. The derived rate constants, *k_c_*, obey a rate law that is first order in [MT_4_] and depend on the specific nature of the catalyst in the order AuT_4_ > CoT_4_ > MnT_4_ > ZnT_4_. Both resonance light scattering (RLS) intensity and extinction in the aggregated samples increase on increasing [MT_4_]. With the exception of AuT_4_, the final aggregated samples obtained at the highest catalyst concentration exhibit a negative Cotton effect in the J-band region, evidencing the occurrence of spontaneous symmetry breaking. The role of the nature of the metal derivative in terms of overall charge and presence of axial groups will be discussed.

## 1. Introduction

Spontaneous self-assembling of molecular units into ordered and larger stable arrangements through non-covalent interactions is a topic that in the last few years has received significant attention from the research community due to its large impact on many aspects of chemistry [1,2]. Porphyrins certainly represent a class of compounds that are extremely interesting in this regard because, depending on both their electronic and steric characteristics, they are susceptible to aggregation forming dimers and/or larger molecular oligomers [3,4,5,6]. In this respect, water-soluble porphyrins are excellent candidates to explore the aggregation process, which can be suitably modulated by varying the medium properties, such as pH and ionic strength, or by taking advantage of the coordination chemistry properties of the central macrocycle. The latter may be indeed readily metallated with a wide variety of metal ions, thereby giving access to peculiar photophysical and photochemical properties [7,8,9,10]. However, despite many reports on the formation of porphyrin homo-aggregates, there are relatively few literature investigations on hetero-aggregated systems. Self-assembly between cationic and anionic meso-substituted porphyrins, mainly driven by electrostatic attraction of peripheral substituent groups, hydrophobic interactions and axial coordination, represents an easy way to design hetero-aggregated supramolecular species [11,12,13,14,15,16,17], even if the formation of discrete hetero-dimers has been reported only in water/organic solvents mixtures [18,19]. In this context, the targeted use of anionic porphyrins such as 5,10,15,20-tetrakis(4-sulfonatophenyl) porphyrin TPPS_4_, whose diacid form is able to give both J- (edge-to-edge) and H-type (face-to-face) self-aggregates, is particularly attractive since these structures exhibit peculiar emergent photophysical properties tunable through aggregate topology and molecular arrangement [20,21,22,23,24,25,26,27,28,29,30]. In the literature, some examples concerning the use of TPPS_4_ in the formation of hetero-aggregated systems have been reported [31,32,33,34,35,36,37] and, among them, some focus on the possibilities of using TPPS_4_ in applicative fields. Shelnutt et al. [35] described the formation of nanotubular J-aggregates whose structure retains own individual porphyrin properties as photocatalytic reduction of metals, while Oztek et al. reported on the potential use of hetero-aggregates as hydrogen storage materials [36].

**Scheme 1:**
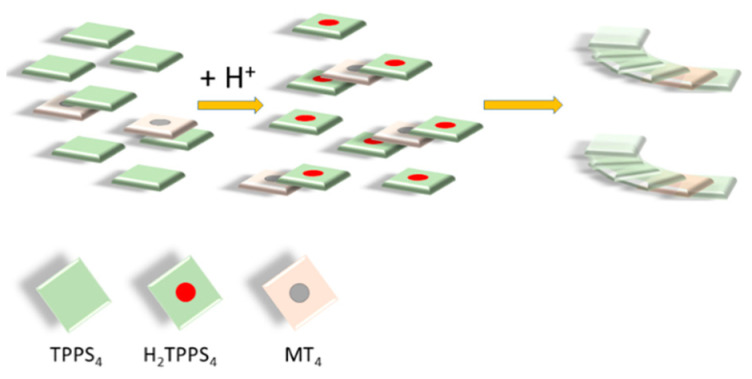
Pictorial sketch of TPPS_4_ protonation and subsequent J-aggregation in the presence of cationic metallo-porphyrins MT_4_.

Other relevant examples in the field involve the additional use of suitable chiral templating units aimed to obtain aggregates exhibiting a well-defined stoichiometric ratio that are able to memorize and amplify the chiral information [33,34,38,39,40,41]. Despite the many reports on thermodynamic and spectroscopic characterization of these supramolecular species, kinetic studies are rather scarce due to the difficulty in controlling the various parameters that influence the aggregation pathways and eventually the final structures at the nano- or mesoscale [42,43]. Notwithstanding, the knowledge of the kinetic behavior is a prerequisite to understanding the factors controlling the rate determining step and the early stages in these complex phenomena. On the basis of our previous spectroscopic investigations on the formation of hetero-oligomers and the subsequent J-aggregates induction [31], here, we describe a detailed kinetic study on the role of cationic metallo-porphyrins MT_4_ in the supramolecular assembling process leading to J-aggregates of TPPS_4_ (Scheme 1). Both MT_4_ and TPPS_4_ porphyrins do not show any tendency towards self-aggregation in the adopted experimental conditions and we anticipate that: (i) the addition of MT_4_ to solutions of TPPS_4_ easily promotes J-aggregation, even under mild acidic conditions; (ii) the kinetic rates and the extent of aggregation depend linearly on the cationic porphyrin concentration and on the overall characteristics of these metal derivatives (charge and axial substituent groups), following the order AuT_4_ > CoT_4_ > MnT_4_ > ZnT_4_; (iii) in most cases, the number of porphyrins in the critical nucleus is slightly less than 3-4, as previously reported for the uncatalyzed process; and iv) except for the most effective metal derivative (AuT_4_), small CD spectra are always detectable, thus adding to the growing number of examples of spontaneous symmetry breaking in J-aggregates [44,45,46,47,48].

## 2. Results and Discussion

**Figure 1:**
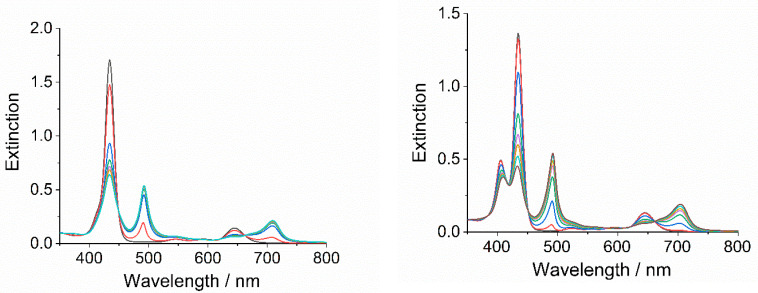
UV/Vis extinction spectral changes for the aggregation of TPPS_4_ into J-aggregates in the presence of CoT_4_ (**left**) and AuT_4_ (**right**) after the addition of hydrochloric acid. For CoT_4_: 0 (black), 40 (red), 80 (blue), 120 (green), 160 (violet), 210 (yellow), 340 s (cyano); For AuT_4_: 0 (black), 10 (red), 20 (blue), 30 (green), 40 (violet), 50 (yellow), 80 (cyano), 220 s (brown). (Experimental conditions: [TPPS_4_] = 3 μM; [MT_4_] = 1.5 μM; [HCl] = 0.00158 M, T = 298 K, total acquisition time 400 s).

At neutral or alkaline pH, aqueous solutions of the parent free-base porphyrin at micro-molar concentration stabilize toward self-aggregation. The nitrogen atoms at the core of the tetra-anionic porphyrin TPPS_4_ are protonated (pKa = 4.9 [49]) and this species is fully converted to its diacid dianionic form H_2_TPPS_4_ at pH 2.8 (B-band at 434 nm, see black line in Figure 1). In the presence of only HCl at this pH, the formation of J-aggregates of H_2_TPPS_4_ is kinetically very slow and a tiny concentration can be detected after at least 24 h. In order to investigate the catalytic role of the various metal derivatives of the cationic tetrakis(4-*N*-methylpyridinium)porphyrin (MT_4_), these latter species have been pre-incubated at different molar ratios R with TPPS_4_ in water (R = [TPPS_4_]/[MT_4_], with R spanning in the range 2 ÷ 30) and then aggregation has been triggered by lowering the pH to 2.8 through addition of HCl. All the investigated MT_4_ derivatives form hetero-aggregates with the anionic TPPS_4_ and their spectroscopic features and stoichiometry have been reported in literature [31]. Figure 1 displays the typical time evolution of the UV/Vis spectra observed for both CoT_4_ (Figure 1, left) and AuT_4_ (Figure 1, right) at the highest ratio R = 2. The gold(III) metal complex interacting with H_2_TPPS_4_ exhibits an initial B-band at 405 nm that decreases in intensity and undergoes a further bathochromic shift to 411 nm, matched by the decrease of the B- and Q-bands of the diacid H_2_TPPS_4_ at 434 and 644 nm, respectively, and the concomitant increase of the J-aggregate B-band at 491 nm, together with its Q-band at 704 nm. MnT_4_ display a B-band at 462 nm and it undergoes a bathochromic shift of 6 nm upon aggregation (see Supporting Information, Figure S7). In the case of CoT_4_, the B-band of this species (434 nm) is under the envelope of the diacid H_2_TPPS_4_ and consequently, not detectable. ZnT_4_ exhibits a B-band at 436 nm and follows the same pattern of CoT_4_. Upon addition of HCl, a decrease of the B-band of the diacid species also occurs, while the J-band at 490 nm increases in intensity.

**Figure 2:**
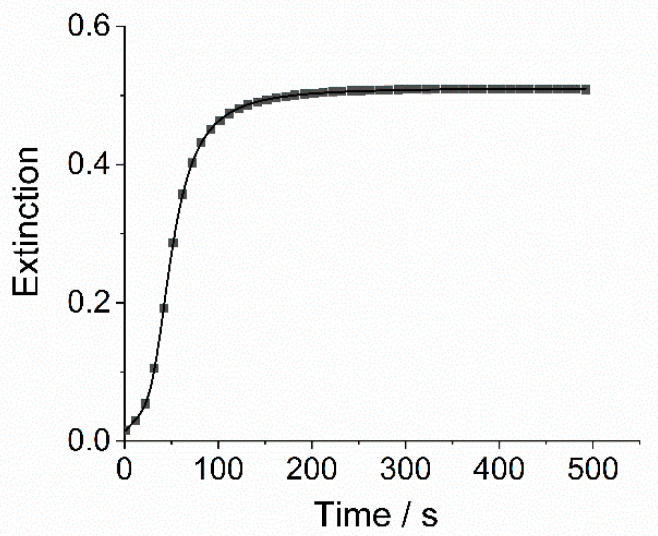
Typical kinetic profile of extinction at 491 nm vs. time for the aggregation of TPPS_4_ catalyzed by CoT_4_. The black line is the best fit of the experimental data to Equation (1) (Experimental conditions: [TPPS_4_] = 3 μM; [CoT_4_] = 1.5 μM; [HCl] = 0.00158 M, T = 298 K).

The kinetics of growth of J-aggregates can be easily followed by monitoring the extinction of the samples at 490 nm. Figure 2 shows a typical kinetic profile for the aggregation induced by CoT_4_. As already reported for acid-induced aggregation of H_2_TPPS_4_, a sigmoidal behavior is clearly observed with a quite short initial incubation or lag-time followed by an exponential growth. These data have been treated by a well-established autocatalytic model, in which two independent pathways are operative: (i) an uncatalyzed one, controlled by a rate k_0_ and (ii) a catalyzed one, dominated by a stretched exponential form with *k_c_* as the rate constant and n as the time exponent. The rate determining step of the second pathway is the initial formation of a nucleus containing m monomeric porphyrin units [50,51]. All the kinetic parameters have been obtained by a non-linear best-fitting procedure that was applied to the experimental extinction data and collected in Table S1 (see Supporting Information), with the exception of k_0_, which makes no or only a minor contribution to the overall fit of the data. 

**Figure 3:**
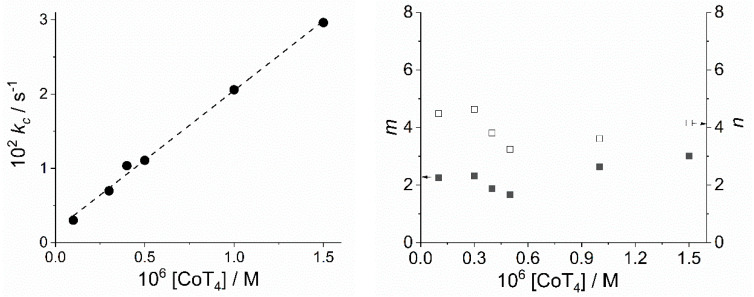
Plot of the autocatalytic rate constants *k_c_* (s^−1^) (**left**), the line represents the linear fit of the experimental data to the equation *k_c_* = (1.7 × 10^−3^ ± 5 × 10^−4^) + (1.88 × 10^−2^ ± 6 × 10^−4^) × [CoT_4_], and the values of m (full squares) and n (empty squares) for the aggregation of TPPS_4_ as a function of [CoT_4_] (**right**). (Experimental conditions: [TPPS_4_] = 3 μM; [HCl] = 0.00158 M, T = 298 K).

As an example, Figure 3 (left) displays the typical linear dependence of the values of the rate constant *k_c_* as a function of the increasing concentration of [CoT_4_]. The related values for the time exponent n range between 3 and 4, while the size of the initial nucleus m ≈ 2–3 (dimer or trimer) is slightly lower with respect to what is reported in the literature for acid-induced aggregation of the parent porphyrin. In these latter cases, m values suggested the involvement of a trimer or a tetramer in the rate determining step [47,52,53,54]. This difference could be ascribed to the different nature of the starting building block for the aggregation process, where a heterodimer stabilized by electrostatic interactions could be responsible for the seeding of the growth. As already reported in the literature [31], the cationic porphyrins investigated here form such kinds of supramolecular species with variable stoichiometry. This observation, together with a certain degree of variation on m values measured for ZnT_4_ and AuT_4_ (see Table S1), suggests the potential formation of concentration dependent hetero-oligomers as initiators for the catalytic growth of the H_2_TPPS_4_ J-aggregates. 

**Figure 4:**
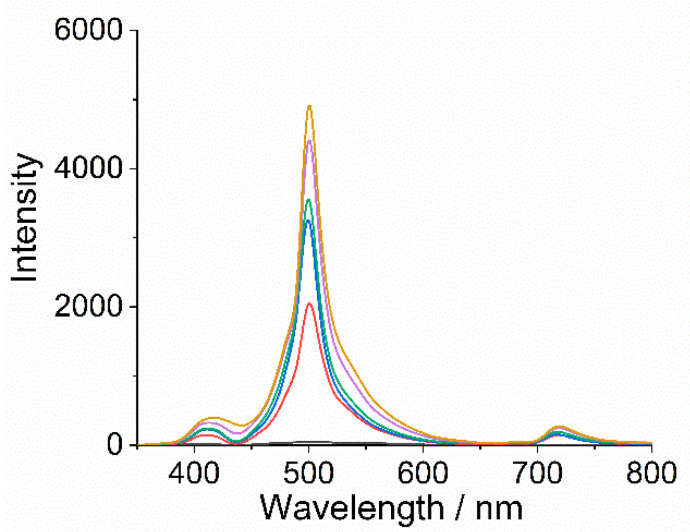
Resonance light scattering (RLS) spectra of the samples at the end of the aggregation process catalyzed by CoT_4_ at different concentrations of this metal derivative. (Experimental conditions: [TPPS_4_] = 3 μM; [HCl] = 0.00158 M, [CoT_4_] = 0.1 μM (black), 0.3 μM (red), 0.4 μM (blue), 0.5 μM (green), 1 μM (violet), 1.5 μM (yellow), T = 298 K).

Resonance light scattering from aggregated samples confirms the presence of quite large arrays of electronically coupled porphyrin units (N > 25) [55]. The intensity of a resonance light scattering (RLS) peak depends on the scattering and the absorption cross-sections of the aggregates and therefore on their size and concentration [56]. Figure 4 shows the RLS spectra of aggregated samples for the case of the CoT_4_ catalyzed process. The intensities of the peaks centered at about 490 nm increase on increasing [CoT_4_]. 

**Figure 5:**
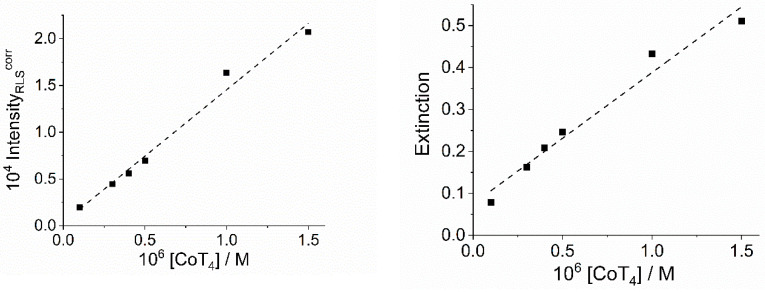
Intensity of RLS spectra corrected for extinction (**left**) and extinction of the samples at the end of the TPPS_4_ aggregation process catalyzed by CoT_4_ (**right**) as a function of the concentration of this metal derivative. (Experimental conditions: [TPPS_4_] = 3 μM; [HCl] = 0.00158 M, T = 298 K). The lines represent the linear best fits to the experimental data (I_RLS_^corr^ = 10^4^ × (0.035 ± 0.074) +10^4^ × (1.419 ± 0.093) × [CoT_4_]; Ext = (0.075 ± 0.022) + (0.313 ± 0.028) × [CoT_4_]).

Analogously, both the intensity of RLS corrected for the extinction of the solutions and the extinction increase linearly on increasing [CoT_4_] (Figure 5), in line with an increment in size and concentration of the formed aggregates. Since the enhancement of the scattered light at resonance with the absorption feature reflects the strong electronic coupling among the porphyrins and increases with their numbers in the aggregate [55], this evidence points to a progressive stabilization of the nano-assemblies mediated by the cationic porphyrins.

**Figure 6:**
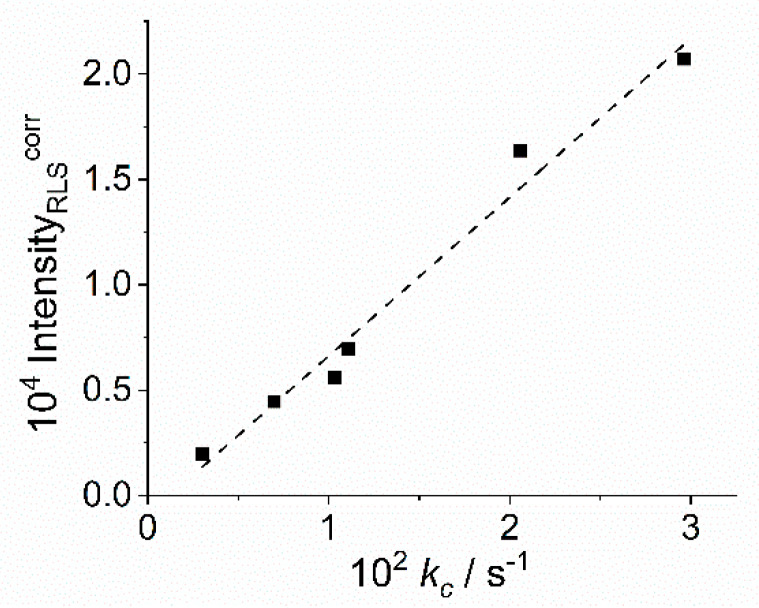
Plot of the RLS intensity at maxima corrected for the extinction of the samples as a function of the corresponding rate constants *k_c_* for the J-aggregation catalyzed by CoT_4_. (Experimental conditions: [TPPS_4_] = 3 μM; [CoT_4_] = 1.5 μM; [HCl] = 0.00158 M, T = 298 K). The line represents the linear best fit to the experimental data (I_RLS_^corr^ = 10^5^ × (−0.009 ± 0.009) + 10^5^ (7.531 ± 0.556) × *k_c_*).

Interestingly, the intensity of RLS increases on increasing the aggregation rate constant *k_c_* (Figure 6). The data have been analyzed through a linear best fitting, obtaining I_RLS_^corr^ = (−912 ± 905) + 10^5^ × (7.53 ± 0.56) × *k_c_*. On one hand, this behavior is in contrast with that observed for the acid-induced aggregation of TPPS_4_, where an inverse dependence has been reported for the growth of porphyrin nanotubes [47]. On the other hand, when these nanoassemblies are formed in the presence of a high concentration of Zn^2+^, again a linear dependence between these two parameters has been reported [52]. These findings suggest that the structural arrangement of the chromophores in these specific supramolecular species are quite different with respect to that involved in nanotubes. Further evidence for a difference in the aggregate architecture stems from the slight broadening of the J-band for all the final aggregated samples. These spectral features appear generally larger than those reported for systems where the Frenkel exciton model for the electronic coupling among chromophores is operative [57].

**Figure 7:**
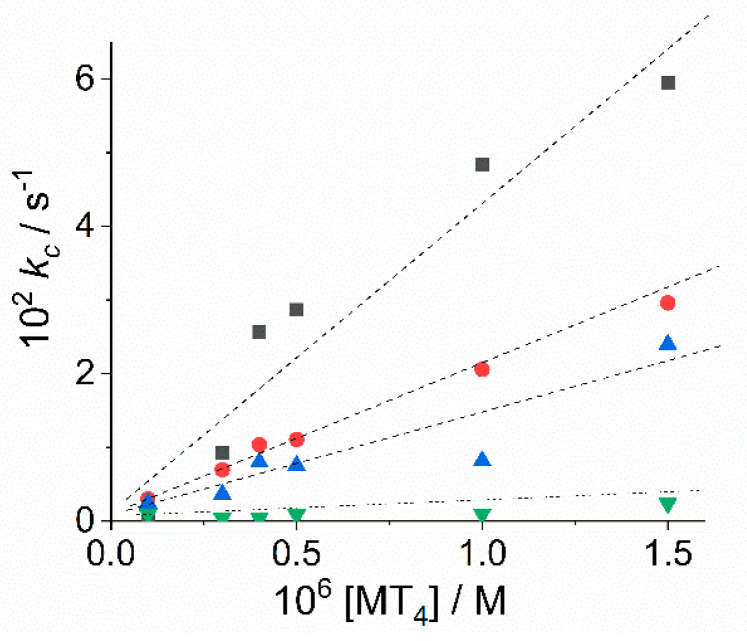
Plot of the autocatalytic rate constants *k_c_* (s^−1^) for the aggregation of TPPS_4_ as a function of [MT_4_] for the various metal derivatives. AuT_4_ (black squares), CoT_4_ (red circles), MnT_4_ (blue triangles), ZnT_4_ (green triangles). (Experimental conditions: [TPPS_4_] = 3 μM; [HCl] = 0.00158 M, T = 298 K). The straight lines represent the linear fit to the rate data, according to the equation *k_c_* = *k*’*_MT_*_4_ × [MT_4_].

**Figure 8:**
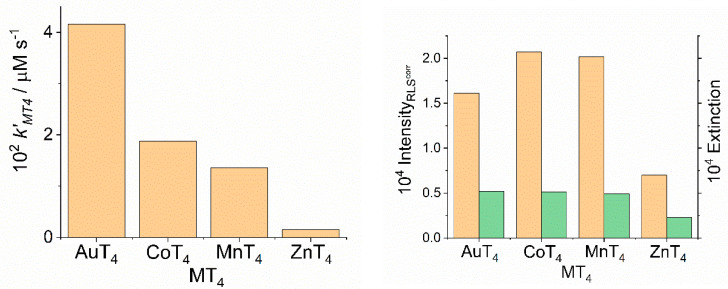
Bar plot of the rate constants *k’_MT_*_4_ (μM × s^−1^) obtained as slope from Figure 7 (**left**) and the RLS intensity at maxima corrected for the extinction (orange bars) and extinction (green bars) of the samples after complete aggregation of TPPS_4_ for the various metal derivatives MT_4_ (**right**). (Experimental conditions: [TPPS_4_] = 3 μM; [MT_4_] = 1.5 μM; [HCl] = 0.00158 M, T = 298 K).

When comparing the values of *k_c_* for the various metal derivatives, a monotonic increase is well evident together with a different efficiency in promoting the aggregation of TPPS_4_. Figure 7 reports the behavior of *k_c_* as a function of increasing [MT_4_] for the investigated cationic porphyrins. All the data have been best-fitted to the equation *k_c_* = *k*’*_MT_*_4_ × [MT_4_] and the values of the relative slope *k*’*_MT_*_4_ follow the trend: AuT_4_ > CoT_4_ > MnT_4_ > ZnT_4_ (Figure 8, left). The ability of the various porphyrins in accelerating the formation of J-aggregates is paralleled by their attitude in stabilizing larger aggregates or higher concentrations, as shown in Figure 8 (right), where the values of I_RLS_^corr^ and extinction for fully aggregated samples at the highest investigated concentration are displayed.

**Figure 9:**
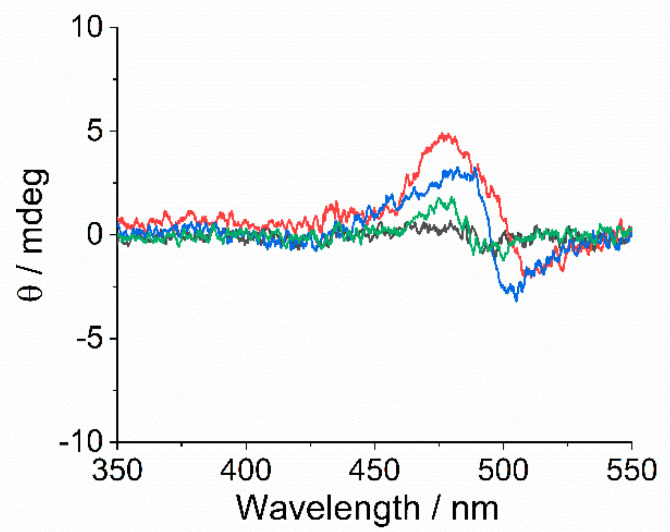
CD spectra of the samples after complete aggregation of TPPS_4_ for the various metal derivatives MT_4_ (AuT_4_ (black), CoT_4_ (red), MnT_4_ (blue), ZnT_4_ (green). (Experimental conditions: [TPPS_4_] = 3 μM; [MT_4_] = 1.5 μM; [HCl] = 0.00158 M, T = 298 K).

An intriguing property of TPPS_4_ J-aggregates is their propensity to spontaneous symmetry breaking, leading to an unbalance in the mixture of enantiomorphous nanostructures. This occurrence can be detected through circular dichroism spectroscopy and these supramolecular assemblies exhibit exciton coupled CD spectra at the wavelength of their absorption bands. In the case of J-aggregates formed in the presence of inorganic acids, the intensity of the usually observed positive Cotton effect can be quite large and an inverse dependence on the aggregation rate has been reported [46,47]. In the present case, very weak negative exciton split CD spectra are detectable at the J-band for all the samples obtained in the presence of the cationic porphyrins, with the exception of AuT_4_ (Figure 9). This evidence supports the hypothesis that the presence of MT_4_ species promotes structurally different aggregates with respect to nanotubes.

**Scheme 2:**
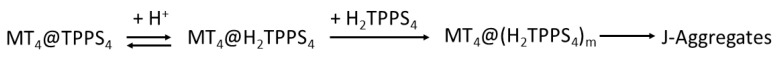
Proposed mechanism for J-aggregation of TPPS_4_ in the presence of cationic metallo-porphyrins MT_4_ under mild acidic conditions.

On the basis of the collected evidence, a mechanistic pathway to aggregation can be proposed (Scheme 2). In water at neutral pH, the TPPS_4_ porphyrin is not protonated and therefore it is a tetra-anion able to strongly interact with cationic MT_4_ metallo-porphyrins, leading to different hetero-aggregates (MT_4_@TPPS_4_). These latter species possess an overall charge that depends on the coordinated metal ion (Au(III) +5; Co(III) +5; Mn(III) +5; Zn(II) +4) in the cationic species. Further, the nature of the metal ion imposes different coordination numbers and geometries: (i) Au(III) 4-coordinated and square planar; (ii) Co(III) and Mn(III) 6-coordinated and octahedral; and (iii) Zn(II) 5-coordinated and square pyramidal. The resulting different charge and geometrical structures of the various metal complexes have an impact on the stoichiometry and interaction among porphyrins in the formation of the hetero-aggregated species. Moreover, upon lowering the pH to 2.8, the TPPS_4_ porphyrin is further protonated at the macrocyclic core and its overall charge decreases to −2 in the diacid H_2_TPPS_4_. This event determines a rearrangement of the hetero-aggregates (MT_4_@H_2_TPPS_4_) and their stoichiometry were reported as ranging from 1:3 to 2:3 for the AuT_4_@H_2_TPPS_4_ species and 2:3 for MnT_4_@H_2_TPPS_4_ and ZnT_4_@H_2_TPPS_4_ [31]. The preference for an edge-to-edge interaction (J-type) in these hetero-oligomers was explained (i) in terms of a steric role of the axial substituent groups, as in the penta- (ZnT_4_) and hexa-coordinated (CoT_4_ and MnT_4_) complexes, thus preventing a potential face-to-face contact (H-type) and (ii) the net positive charge at the core of the macrocycle (AuT_4_), leading to a repulsive interaction with the diprotonated H_2_TPPS_4_. All these factors determine the observed trend in the efficiency of the MT_4_ species to influence both the rates and the eventual extent of aggregation. Indeed, these J-hetero-oligomers can be considered as the real initiators of the assembling process. Alternatively, the further addition of H_2_TPPS_4_ monomers determines the formation of the critical nucleus (MT_4_@(H_2_TPPS_4_)_m_). From this point on, the autocatalytic pathway controlled by the rate constants *k_c_* leads to the growth of the final J-aggregates. It is interesting to observe that, when detectable, the final position of the B-band related to the MT_4_ species in the UV/Vis spectra suggests their involvement into the nano-assemblies (Figure S7). In addition, slight bathochromic shifts can be measured in the B-and Q-bands of the final J-aggregates (AuT_4_, 492/704 nm; CoT_4_, 492/709 nm; MnT_4_ 493/709 nm; ZnT_4_, 496/712 nm), supporting the hypothesis of a substantial incorporation of the cationic metallo-porphyrins in the J-aggregates. This evidence is in line with literature data showing the incorporation of Sn(IV) cationic species into nanotubes of H_2_TPPS_4_ [35].

## 3. Materials and Methods

Materials. 5, 10, 15, 20-tetrakis(4-sulfonatophenyl)porphine (TPPS_4_) was purchased from Aldrich Chemical Co. Au(III), Mn(III), Co(III), Zn(II) derivatives of tetrakis(4-*N*-methylpyridyl)porphine (H_2_T_4_) were prepared according to literature procedures [7,8,9,10]. These porphyrins were solubilized in high-purity doubly distilled water from Galenica Senese. The range of concentration used in our experiments was determined spectrophotometrically using the molar extinction coefficients at the Soret maxima (TPPS_4_: 5.33 × 10^5^ M^−1^cm^−1^, λ = 414 nm; AuT_4_: 2.82 × 10^5^ M^−1^cm^−1^, λ = 403 nm; MnT_4_: 1.29 × 10^5^ M^−1^cm^−1^, λ = 462 nm; CoT_4_: 1.68 × 10^5^ M^−1^cm^−1^, λ = 434 nm; ZnT_4_: 1.81 × 10^5^ M^−1^cm^−1^, λ = 436 nm).

Methods. UV/Vis absorption spectra and kinetic traces were measured on an Agilent model HP 8453 diode array spectrophotometer. An UV filter (Hoya glass type UV-34, cut-off: 340 nm) was used in the kinetic measurements in order to cut off the UV component of the spectrophotometer lamp, preventing the photodegradation of porphyrins. Resonance light scattering (RLS) experiments were performed on a Jasco mod. FP-750 spectrofluorimeter, adopting a synchronous scan protocol with a right angle geometry [56]. Circular dichroism experiments were carried out on a Jasco mod. J-710 spectropolarimeter. Aggregation occurs by addition of a known volume of a concentrated stock solution of hydrochloric acid (final concentration [HCl] = 1.58 mM, pH = 2.8) to a solution of porphyrin (3 µM) premixed with a solution of metal cationic porphyrins (final concentration [MT_4_] = 0.1, 0.3, 0.4, 0.5, 1, 1.5 µM). Kinetic experiments were run by acquiring extinction spectra from the mixed solutions placed in the thermostatic sample holder of the spectrophotometer (298 K). The aggregation kinetics exhibit a sigmoidal profile that can be fitted by an autocatalytic model [47,50,51]. The extinction traces collected at 490 nm were analyzed by a non-linear least square fit to the equation:

Ext_t_ = Ext_∞_ + (Ext_0_ − Ext_∞_) (1 + (*m* – 1){*k*_0_t + (*n* + 1)^−1^ (*k_c_* t)*^n^*^+1^})^−1/(*m*−1)^ (1)

where Ext_0_, Ext_∞_, *k*_0_, *k_c_*, m, and n are the optimized parameters.

## 4. Conclusions

Kinetic investigations offer the opportunity to gain insights on how the growth of supramolecular assemblies is controlled by specific changes of the medium and other external factors. The J-aggregates formed by the water sulfonated porphyrin TPPS_4_ have been highly investigated for their ability to self-assemble into a variety of nano- and mesoscopic supramolecular structures. The specific arrangement and electronic coupling of the chromophores inside these species control their spectroscopic features and properties, such as their chirality. Understanding the role of other chemical components able to mediate, catalyze, and eventually to be incorporated into the final aggregates is therefore very important. The present study adds another small piece of information on these systems. Here, we demonstrated that MT_4_ porphyrins are effective in accelerating the J-aggregation of H_2_TPPS_4_ and that their efficiency is strongly dependent on their electronic and steric characteristics. Cationic metallo-porphyrins enter as part of the nano-architecture and the presence of transition metal ions could potentially introduce novel properties, such as catalytic activity.

## Supplementary Materials

The following are available online. Figure S1: Autocatalytic rate constants *k_c_* and the values of *m* and *n* for the aggregation of TPPS_4_ vs. [AuT_4_], Figure S2: RLS intensity and extinction vs. [AuT_4_], Figure S3: Autocatalytic rate constants *k_c_* and the values of m and n for the aggregation of TPPS_4_ vs. [MnT_4_], Figure S4: RLS intensity and extinction vs. [MnT_4_], Figure S5: Autocatalytic rate constants *k_c_* and the values of m and n for the aggregation of TPPS_4_ as function of [ZnT_4_], Figure S6: RLS intensity and extinction vs. [ZnT_4_], Figure S7: UV/Vis extinction spectra of TPPS_4_ J-aggregates in the presence of AuT_4_, CoT_4_, ZnT_4_ and MnT_4_, Table S1: Kinetic parameters for TPPS_4_ aggregation vs. [MT_4_].
