# Supplementary material for: Kinetic Investigation on Tetrakis(4-Sulfonatophenyl)Porphyrin J-Aggregates Formation Catalyzed by Cationic Metallo-Porphyrins
Source: Molecules. 2020 Dec 5;25(23):5742. doi: 10.3390/molecules25235742 (PMC7731138; doi:10.3390/molecules25235742)
Supplement: Supplementary file 1 [file molecules-25-05742-s001.pdf]

Supporting Information

for

Kinetic investigation on tetrakis(4-sulfonatophenyl)porphyrin J-aggregates formation catalyzed by cationic metallo-porphyrins

Ilaria Giuseppina Occhiuto[1], Roberto Zagami[2], Mariachiara Trapani[2], Maria Angela Castriciano[2], Andrea Romeo[1,2], Luigi Monsù Scolaro[1,2]*

[1] Dipartimento di Scienze Chimiche, Biologiche, Farmaceutiche ed Ambientali, University of Messina and C.I.R.C.M.S.B V.le F. Stagno D'Alcontres, 31 - 98166 Messina, Italy

[2] CNR - ISMN Istituto per lo Studio dei Materiali Nanostrutturati c/o Dipartimento di Scienze Chimiche, Biologiche, Farmaceutiche ed Ambientali, University of Messina, V.le F. Stagno D'Alcontres, 31 - 98166 Messina, Italy

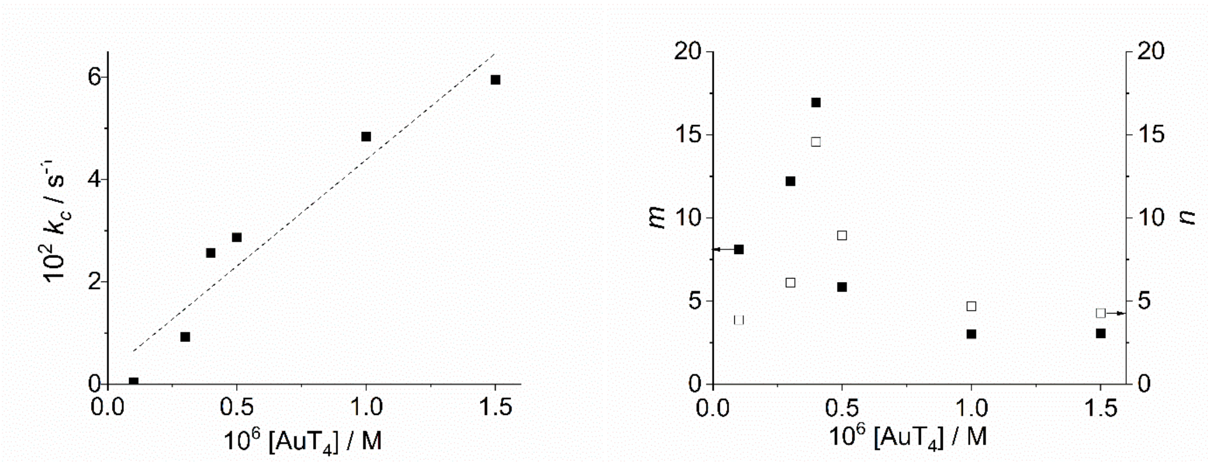

Figure S1. Plot of the autocatalytic rate constants k_c (s^{-1}) (left), and the values of m (full squares) and n (empty squares) for the aggregation of $TPPS_4$ as function of [AuT_4] (right). (Experimental conditions: [$TPPS_4$] = 3 μM; [HCl] = 0.00158 M, T = 298 K). The lines represent the linear best fits to the experimental k_c data to the equation: $k_c = (2.34 \pm 4.72) \times 10^{-3} + (4.15 \pm 0.60) \times 10^{-2} \times [AuT_4]$.

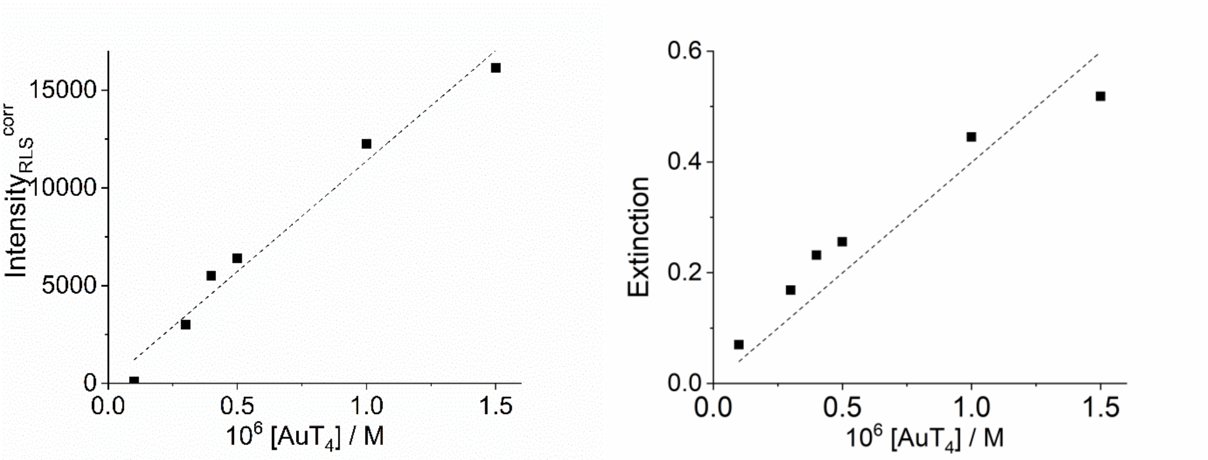

Figure S2. Intensity of RLS spectra corrected for extinction (left) and extinction of the samples (right) at the end of the $TPPS_4$ aggregation process catalyzed by AuT_4 as function of the concentration of this metal derivative (Experimental conditions: [$TPPS_4$] = 3 μM; [HCl] = 0.00158 M, T = 298 K). The lines represent the linear best fits to the experimental data (a: $I_{RLS}^{corr} = 10^4 \times (0.071 \pm 0.071) + 10^4 \times (1.130 \pm 0.089) \times [AuT_4]$; b: $Ext = (0.005 \pm 0.002) + (0.399 \pm 0.033) \times [AuT_4]$).

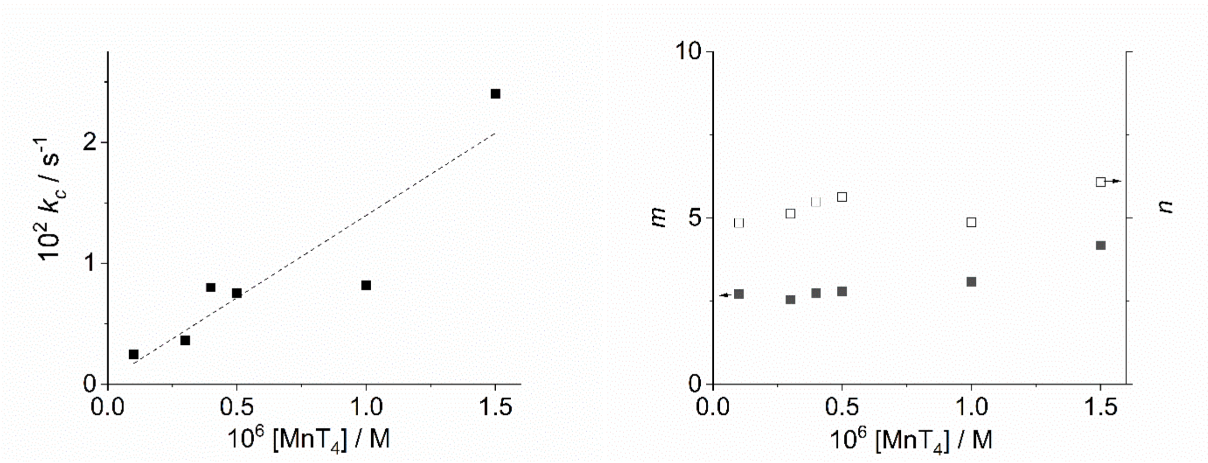

Figure S3. Plot of the autocatalytic rate constants k_c (s^{-1}) (left), and the values of m (full squares) and n (empty squares) for the aggregation of $TPPS_4$ as function of [MnT_4] (right). (Experimental conditions: [$TPPS_4$] = 3 μM; [HCl] = 0.00158 M, T = 298 K). $k_c = (0.36 \pm 2.41) \times 10^{-3} + (1.36 \pm 0.30) \times 10^{-2} \times [MnT_4]$.

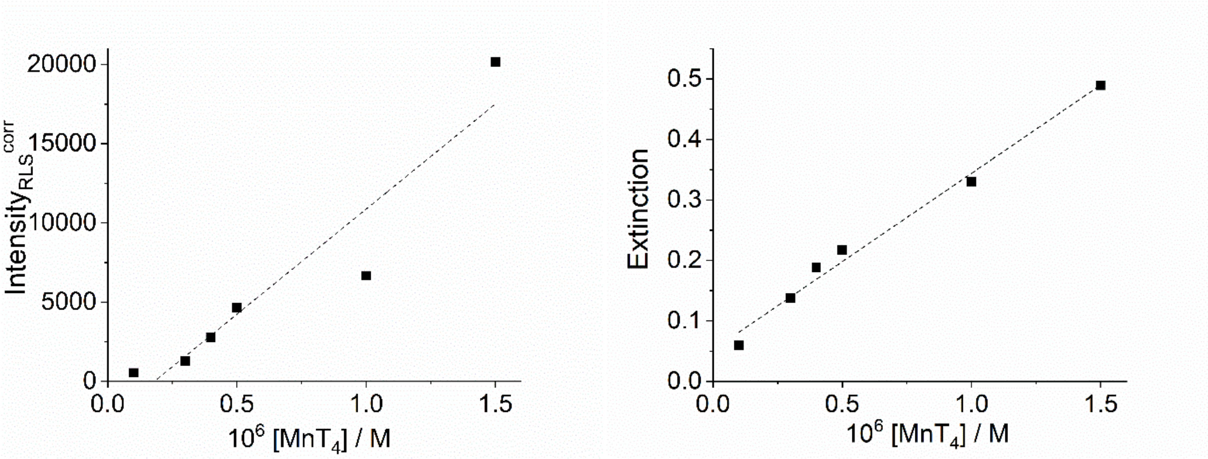

Figure S4. Intensity of RLS spectra corrected for extinction (left) and extinction of the samples (right) at the end of the $TPPS_4$ aggregation process catalyzed by MnT_4 as function of the concentration of this metal derivative (Experimental conditions: [$TPPS_4$] = 3 μM; [HCl] = 0.00158 M, T = 298 K). The lines represent the linear best fits to the experimental data (a: $I_{RLS}^{corr} = 10^4 \times (-0.239 \pm 0.179) + 10^4 \times (1.327 \pm 0.226) \times [MnT_4]$; b: Ext = $(0.052 \pm 0.013) + (0.292 \pm 0.016) \times [MnT_4]$).

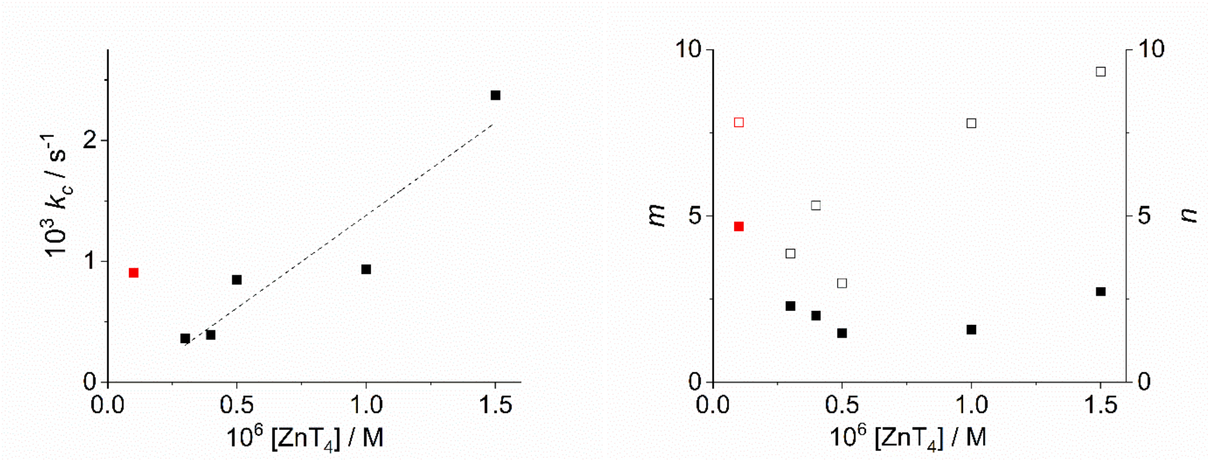

Figure S5. Plot of the autocatalytic rate constants k_c (s^{-1})(left), and the values of m (full squares) and n (empty squares) for the aggregation of $TPPS_4$ as function of [ZnT_4] (right). (Experimental conditions: [$TPPS_4$] = 3 μM; [HCl] = 0.00158 M, T = 298 K). $k_c = (-0.15 \pm 0.281) \times 10^{-3} + (1.53 \pm 0.32) \times 10^{-3} \times [ZnT_4]$.

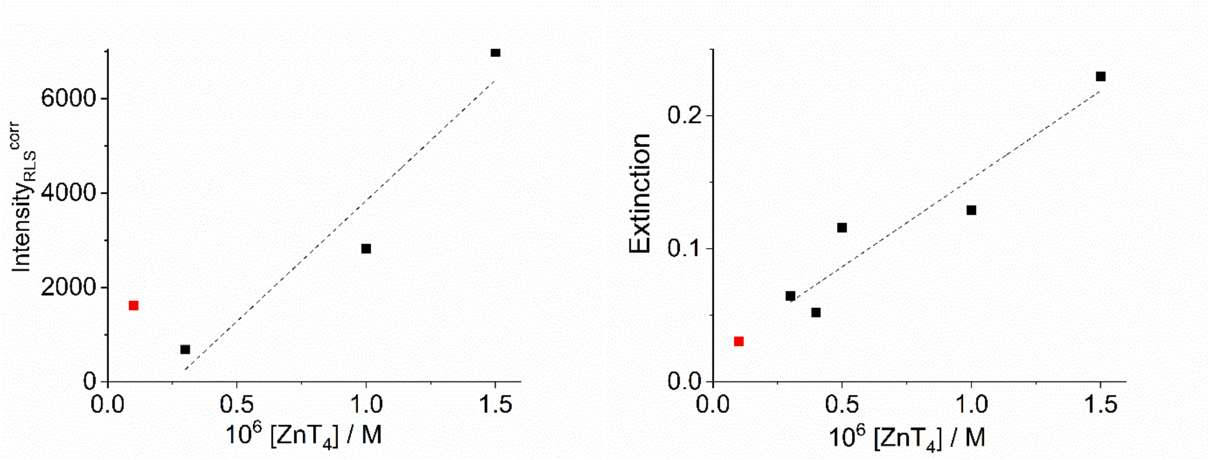

Figure S6. Intensity of RLS spectra corrected for extinction (left) and extinction of the samples (right) at the end of the $TPPS_4$ aggregation process catalyzed by ZnT_4 as function of the concentration of this metal derivative (Experimental conditions: [$TPPS_4$] = 3 μM; [HCl] = 0.00158 M, T = 298 K). The lines represent the linear best fits to the experimental data (a: $I_{RLS}^{corr} = 10^4 \times (-0.127 \pm 0.154) + 10^4 \times (0.511 \pm 0.146) \times [ZnT_4]$; b: $Ext = (0.020 \pm 0.022) + (0.132 \pm 0.026) \times [ZnT_4]$).

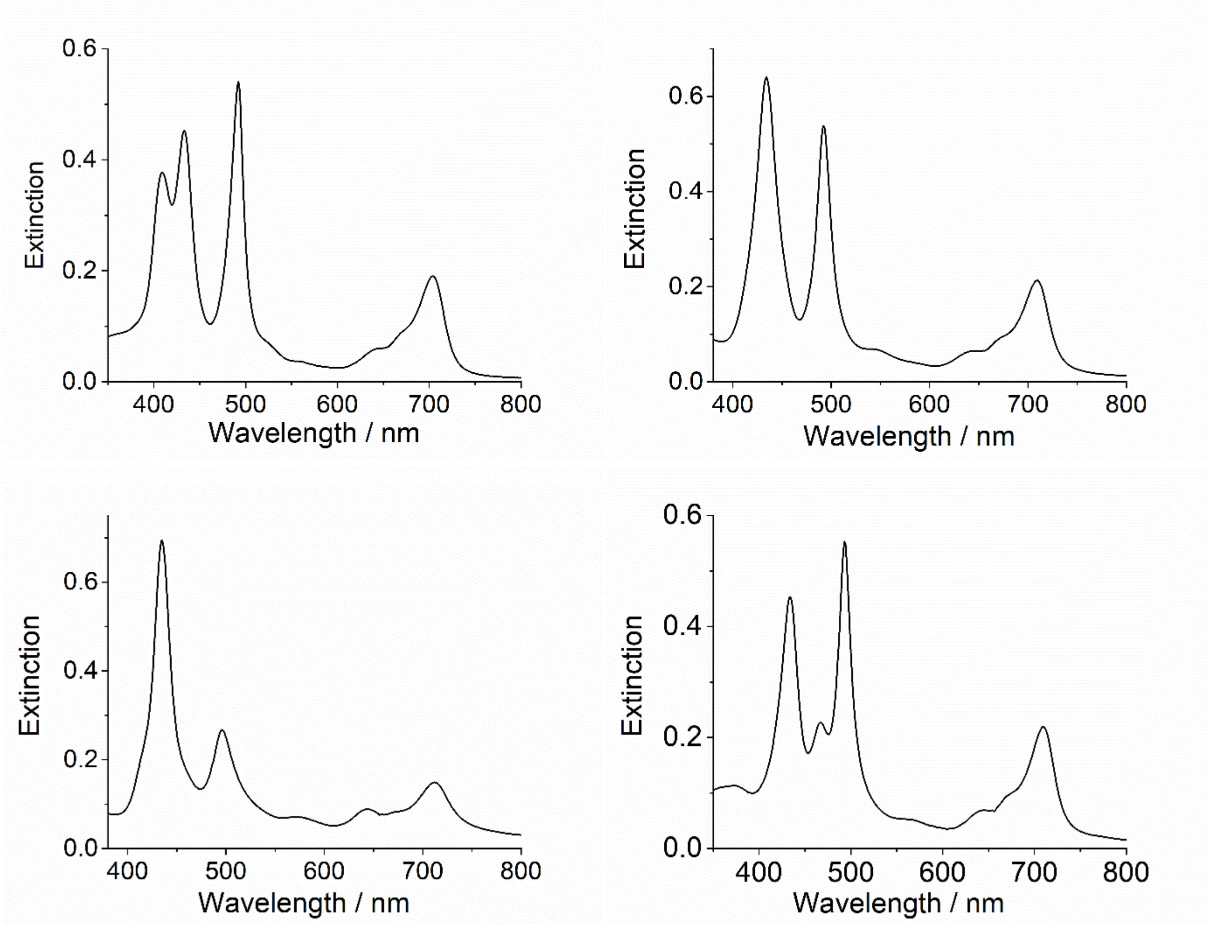

Figure S7. UV/Vis extinction spectra of $TPPS_4$ J-aggregates in the presence of AuT_4 (upper left), of CoT_4 (upper right), of ZnT_4 (lower left), of MnT_4 (lower right). (Experimental conditions: [$TPPS_4$] = 3 μM; [MT_4] = 1.5 μM; [HCl] = 0.00158 M, T = 298 K).

Table S1. Relevant kinetic parameters for $TPPS_4$ aggregation in J-aggregates (k_c, m and n) as function of $[MT_4]$

	$10^6 \times [MT_4]$ / M	$10^3 \times k_c$ / s^{-1}	m	n
**Au**	0.1	0.34 ± 0.02	8.1 ± 0.3	3.9 ± 0.1
	0.3	9.27 ± 0.25	12.2 ± 1.0	6.1 ± 0.5
	0.4	25.7 ± 0.1	16.9 ± 7.2	14.6 ± 6.9
	0.5	28.7 ± 0.1	5.9 ± 1.1	8.9 ± 2.0
	1.0	48.4 ± 0.1	3.0 ± 0.1	4.7 ± 0.2
	1.5	59.5 ± 0.2	3.1 ± 0.1	4.3 ± 0.2
**Co**	0.1	3.02 ± 0.02	2.3 ± 0. 1	4.5 ± 0.2
	0.3	6.92 ± 0.02	2.3 ± 0. 1	4.6 ± 0.1
	0.4	10.3 ± 0.2	1.9 ± 0. 1	3.8 ± 0. 1
	0.5	11.1 ± 0.1	1.7 ± 0.1	3.2 ± 0.1
	1.0	20.6 ± 0.1	2.6 ± 0.1	3.6 ± 0.2
	1.5	29.6 ± 0.1	3.0 ± 0.1	4.2 ± 0.1
**Mn**	0.1	2.46 ± 0.02	2.7 ± 0.1	4.6 ± 0.1
	0.3	3.64 ± 0.02	2.5 ± 0.1	5.1 ± 0.1
	0.4	8.00 ± 0.05	2.7 ± 0.1	5.5 ± 0.3
	0.5	7.56 ± 0.01	2.8 ± 0.1	5.6 ± 0.3
	1.0	8.18 ± 0.07	3.1 ± 0.2	4.9 ± 0.4
	1.5	24.0 ± 0.1	4.1 ± 0.4	6.1 ± 0.7
**Zn**	0.1	0.904 ± 0.009	4.7 ± 0.1	7.8 ± 0.1
	0.3	0.364 ± 0.008	2.3 ± 0.1	3.9 ± 0.1
	0.4	0.394 ± 0.001	2.0 ± 0.1	5.3 ± 0.1
	0.5	0.844 ± 0.021	1.5 ± 0.1	3.0 ± 0.2
	1.0	0.934 ± 0.004	1.6 ± 0.01	7.8 ± 0.01
	1.5	2.37 ± 0.01	2.7 ± 0.1	4.2 ± 0.4

$[TPPS_4]$ = 3 μM; [HCl] = 0.00158 M, T = 298 K
